# Long-Term Behavior and Microstructure of High-Performance Concrete with Coal Slag

**DOI:** 10.3390/ma18112585

**Published:** 2025-06-01

**Authors:** Piotr Smarzewski

**Affiliations:** Faculty of Civil Engineering and Geodesy, Military University of Technology, 2 Gen. Sylwestra Kaliskiego, 00-908 Warsaw, Poland; piotr.smarzewski@wat.edu.pl; Tel.: +48-698695284

**Keywords:** high-performance concrete, coal slag, porosity, water absorption, compressive strength, flexural strength, splitting tensile strength, modulus of elasticity, ultra-pulse velocity, microstructure

## Abstract

Recycling in the construction industry is a necessity, not just a fashionable trend in scientific research. The use of coal slag aggregates in concrete means a significant reduction in environmental footprint and should be a priority. For these reasons, this study presents tests of the physical and mechanical properties of high-performance concrete (HPC) with coal slag (CS) used as a replacement for natural coarse aggregate in the amounts of 10%, 20%, and 30% after a long curing time. The investigation determined the porosity, water absorption, density, compressive strength, flexural strength, tensile splitting strength, modulus of elasticity, and ultrasonic pulse velocity (UPV), and analyzed HPC microstructure at 28, 56 days, as well as 2 years of maturation. The use of coal slag resulted in significant increases in compressive strength, flexural strength, and tensile splitting strength compared to reference concrete. However, for HPC with CS, a slight decrease in the elastic modulus and UPV was obtained. The SEM analysis showed a very good adhesion of the cement paste to the slag aggregate. In general, research shows that it is possible to obtain durable high-performance concrete with a 30% replacement of natural aggregate by coal slag.

## 1. Introduction

High-performance concrete (HPC) is a composite material characterized by compressive strength greater than 60 MPa, typically produced with a high cement content, microsilica, fine sand, coarse aggregate (grain size < 12 mm), water, and superplasticizer admixtures [1]. Its main advantages include reduced component consumption due to smaller and more slender structural dimensions, as well as improved durability and resistance to environmental degradation. These features make HPC particularly suitable for use in demanding structural applications, such as bridges, high-rise buildings, and marine structures [2].

Coarse aggregate significantly influences the quality and durability of concrete. To ensure proper performance, aggregates must be homogeneous in physical properties and free from harmful components. Recently, researchers have increasingly turned to industrial and demolition waste as a substitute for traditional aggregates. These alternative materials can offer technical, environmental, and economic benefits, such as reducing landfill volumes, reducing greenhouse gas emissions, and minimizing the depletion of natural resources. Prior studies have shown that concrete incorporating recycled or waste-derived aggregates can achieve comparable or even superior performance compared to conventional concrete. For example, Meyer [3] outlined the environmental advantages of using industrial byproducts in concrete, noting that slag and fly ash can improve mechanical performance while supporting sustainability goals. Kansal et al. [4] demonstrated that the combined use of silica fume and steel slag improves compressive strength in high-performance concrete incorporating nanosilica. Brescia-Norambuena et al. [5] reported synergistic improvements in mechanical performance, particularly under dynamic loading, when silica fume and nanosilica were used together with polypropylene fibers. Hu et al. [6] investigated the residue of dolomite powder in cement paste, showing enhanced hydration and strength at an early age along with favorable life-cycle performance. Finally, Smarzewski and Błaszczyk [7] confirmed that the addition of cement kiln dust (CKD) improves the long-term compressive and tensile strength of high-performance concrete while refining its microstructure over time. Other studies have highlighted durability improvements in concrete that incorporate various mineral additives and industrial byproducts. Akbulut et al. [8] provided a comprehensive review showing that both Class-C and Class-F fly ash contribute to reduced permeability and improved resistance to chemical attack in cement-based materials. Liu et al. [9] found that incorporating dolomite rock powder and iron tailings powder into cement pastes improved electrical resistivity and microstructure, thereby improving durability against external agents. Barnat-Hunek et al. [10] demonstrated that the hydrophilization of lightweight aggregate concrete with sewage sludge significantly increased surface water resistance and contact angle, indicating improved performance under moisture exposure. Akbulut et al. [11] also reported that lightweight, self-compacting, eco-friendly concrete with fly ash and silica fume exhibited superior thermal insulation and durability features. Furthermore, Liu et al. [12] showed that alkali-activated dolomite dust composites showed improved structural integrity and chemical resistance under harsh environmental conditions. Furthermore, the use of industrial and demolition waste materials has been widely acknowledged for its environmental advantages and alignment with the principles of the circular economy. Sales and De Souza [13] demonstrated that mortars and concretes that incorporate water treatment sludge and demolition rubble not only reduce landfill burden but also maintain satisfactory mechanical properties. McNeil and Kang [14], in a comprehensive review, emphasized that recycled concrete aggregates significantly contribute to resource conservation and CO_2_ emissions reduction, provided quality control is maintained. Pedro et al. [15] further showed that high-performance concrete made with recycled aggregates, combined with fly ash and densified silica fume, exhibited greater durability, supporting the feasibility of long-lasting, sustainable structural materials.

In line with the principles of the circular economy, the European Union has introduced directives encouraging the reuse of industrial byproducts in construction. Among these, the byproducts of coal combustion, including ash, slag, and fluidized bed residues, represent a promising resource for concrete production [16]. The use of such materials not only reduces the need for virgin aggregates but also contributes to the reduction of environmental hazards related to waste storage.

Coal slag is a byproduct with a glassy and chemically stable structure that contains approximately 50% silica and significant amounts of alumina. It exists in two forms, depending on the cooling rate: unburnt slag (dark gray, porous) and burnt slag (brick-red, hard grains) [17]. Its bulk and volumetric densities range from 700 to 1800 kg/m^3^, with porosity up to 60% and water absorption as high as 20%. The suitability for construction use depends on limiting the presence of unburned carbon, sulfates, iron sulfide, and other impurities that may compromise concrete performance [18]. Regulatory frameworks define acceptable thresholds for ignition loss, particle size distribution, foreign matter, and natural radioactivity [19].

Extensive research has been conducted on the incorporation of various types of waste aggregates in ordinary and lightweight concrete. Studies by Bouguerra et al. [20], Steiger and Hurd [21], and others [22,23,24,25,26,27,28,29] have evaluated the effects of different byproducts on the compressive strength, porosity, thermal conductivity, and long-term durability of concrete. Although some of these materials can decrease stiffness or increase porosity, they often improve mechanical strength or chemical resistance, especially when used in combination with pozzolanic additives such as silica fume. However, the behavior of HPC with high levels of coarse aggregate substitution, especially over long curing periods, remains not sufficiently studied.

Furthermore, the fracture behavior and energy dissipation mechanisms of heterogeneous brittle materials, such as coal-bearing rocks or notched geological media, have recently been explored in the context of dynamic loading and high-energy pulse phenomena. Studies on notch geometry and fracture propagation in rocks [30], the distribution of shock wave energy during high-voltage fracturing [31], and energy absorption in gas-bearing coal under impact conditions [32] emphasize the importance of internal structure, microcrack dynamics, and material composition in the management of the long-term mechanical response. These insights, although derived from geotechnical materials, offer conceptual parallels to high-performance concrete that incorporates porous waste aggregates, especially regarding crack propagation, stress concentration, and microstructural evolution under prolonged or intense stress conditions.

However, most previous studies on concrete with industrial byproducts have concentrated primarily on ordinary or lightweight concrete, typically investigating short- or medium-term curing durations (up to 90 days). For example, Bouguerra et al. [20] studied the porosity effects of lightweight aggregates, while Steiger and Hurd [21] focused on mechanical behavior under short-term conditions. Although some investigations (e.g., Thomas et al. [22], Syarif et al. [27]) explored the use of slags and ashes, detailed studies specifically addressing the long-term performance and microstructural evolution of high-performance concrete with coal slag used as coarse aggregate remain limited. In particular, the effects of prolonged curing periods extending beyond one year have not yet been comprehensively documented, leaving significant gaps regarding durability and long-term mechanical behavior.

Although many studies have investigated the use of industrial byproducts in ordinary and lightweight concretes, the long-term performance of high-performance concrete (HPC) incorporating coal slag (CS) as a coarse aggregate remains insufficiently explored. In particular, there is a lack of comprehensive research on how varying levels of CS substitution affect the mechanical properties, microstructure, and durability of HPC, especially after extended curing periods beyond one year.

This study addresses this gap by evaluating the effects of replacing 10%, 20%, and 30% of coarse natural aggregate with coal slag in HPC. The experimental program includes an extended testing regime covering 28, 56, and 730 days and examines physical (porosity, density, water absorption), mechanical (compressive, flexural, splitting tensile strength, elastic modulus), and microstructural properties (SEM/EDX), as well as ultrasonic pulse velocity (UPV).

The paper is structured as follows. Section 2 presents materials and methods, including mix design, experimental procedures, and testing protocols. Section 3 discusses the results related to the physical, mechanical, and microstructural performance of HPC with coal slag at different curing ages, and analyzes the findings in the context of existing literature and highlights the implications of using coal slag as a coarse aggregate in HPC. Finally, Section 4 summarizes the main conclusions and provides directions for future research.

## 2. Research Program

### 2.1. Characteristics of the Materials

Portland cement CEM I 42.5R and compacted microsilica were used to produce high-performance concrete. The physical and chemical parameters of the microsilica were provided by the manufacturer, Stachema. The CEMEX Portland cement of class CEM I 42.5R was defined according to PN-EN 197-1:2012 [33]. The physical and chemical parameters of cement were determined on the basis of PN-EN 196-6:2019-01 [34]. Its strengths were tested according to PN-EN 196-1:2016-07 [35]. The chemical composition and technical parameters of the binding components were determined, and the results are presented in Table 1.

The mineral composition of Portland cement was analyzed by X-ray diffraction (XRD) using the powder method, using an X-ray diffractometer with a goniometer, a copper lamp, and a graphite monochromator. The dominant components of CEM I 42.5R cement are the sum of tetracalcium aluminoferrite with tricalcium aluminate (C_4_AF + 2C_3_A) in the amount of 19.81%, tricalcium aluminate (C_3_A) in the amount of 5.30%, and alkali Na_2_Oeq in the amount of 0.54%. It can be seen that the microsilica in the compacted form used for the production of high-performance concrete meets the ignition loss requirements, which do not exceed 4% by weight, and the specific surface area, which is greater than 15,000 m^2^/kg.

**Figure 1:**
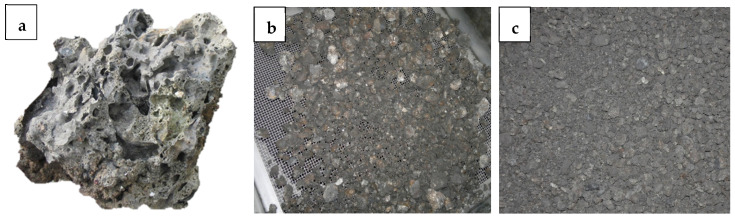
Coal slag (**a**) in natural size, (**b**) during screening, (**c**) with fraction 2–16 after drying.

Dry silica sand with a fraction of 0.05–2 mm was used as the fine aggregate, while gravel aggregate with a fraction of 2–16 mm was used as the coarse aggregate. Sand specific gravity and water absorption were 2.67 and 1.12%, respectively, and gravel aggregate was 2.55 and 0.15%. A lump of natural size of coal slag is shown in Figure 1a. It was initially crushed in the Los Angeles drum and then screened (Figure 1b) to obtain a fraction of 2–16 mm (see Figure 1c). The waste aggregate had a density of 2400 kg/m^3^ and a tapped bulk density of approximately 1080 kg/m^3^.

**Figure 2:**
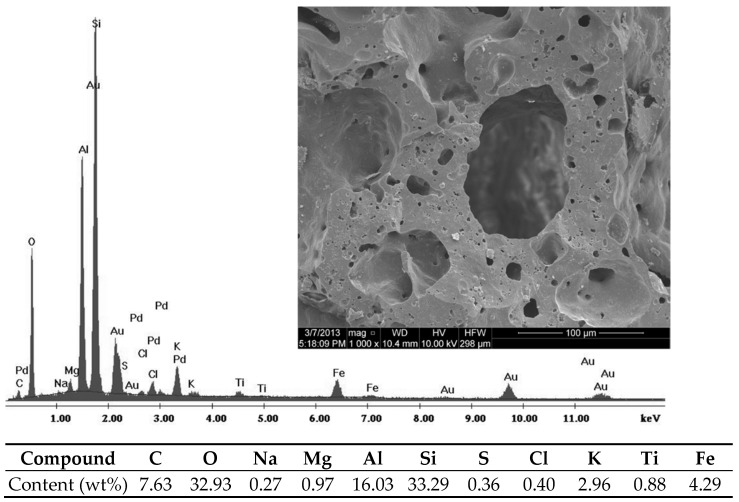
Microstructure of CS, EDX spectrum, and elemental composition.

Morphology, microtopography, and qualitative analysis of chemical composition in the area of the main mineral components of coal slag were determined using a scanning electron microscope (SEM) equipped with a chemical composition analysis system based on dispersive X-ray (EDX). Two slag samples were prepared in the form of thin-layer plates. After drying, the samples were coated with a thin layer of gold and then scanned with an electron microscope with an electron X-ray (EDX) analyzer. The results were developed in the form of microphotographs, spectral graphs, and tables, and are presented in Figure 2.

The mineralogy properties of the slag were determined by the powder method using X-ray diffraction. The dominant component of coal slag (CS) is 50% silica (SiO_2_) in the form of crystallized quartz. Furthermore, CS includes 23.7% alumina (Al_2_O_3_) and 4.9% iron oxide (Fe_2_O_3_). Phases of aluminosilicate (Al_6_Si_2_O_13_) and mullite (Al_2_SiO_5_) were identified with contents, respectively, of 72% and 24%, and ilmenite (FeTiO_3_) in quantity of 4%. Then, the loss of ignition of the coal slag was tested. For this purpose, a small amount of slag was crushed in a mortar. Then the slag sample was weighed to the nearest 0.0005 g (*m*_CS_) and placed in a precalcined and weighed crucible (*m*_m−p_). The crucible with the slag sample was closed with a lid and placed in a muffle furnace heated to 950 ± 25 °C for 30 min. After exposure to high temperature, the crucible was cooled in a desiccator to room temperature. Then, the mass of the slag sample was weighed together with the crucible after ignition (*m*_CS,c_). The following formula was used to determine the ignition loss.

(1) $$\mathrm{loss}\;\mathrm{on}\;\mathrm{ignition}=\frac{m_{CS}-m_{CS,c}}{m_{CS}-m_{m-p}}\times 100\%$$

where

$m_{CS}$—mass of the analytical slag sample with the crucible before calcination (g)

$m_{CS,c}$—mass of the analytical slag sample with the crucible after calcination (g)

$m_{m-p}$—mass of the crucible (g)

The average loss of ignition of the slag determined for three samples was 13.2%.

For the slag sample, the concentrations of natural radioactive isotopes were tested by gamma spectrometry using a three-channel natural radioactive pollutant analyzer [36]. The results, together with the total measurement uncertainties calculated at a 95% confidence level, are presented in Table 2.

The values of the activity indices f_1_ and f_2_ and the dose rate values are f_1_ = 1.09, f_2_ = 122.1 Bq/kg, and m_D_ = 142.4 nGy/h, respectively. It can be seen that the activity index value exceeds the limit value f_1_ = 1, but this increase does not exceed the allowed value of 20% [19]. On the other hand, the value of f_2_ does not exceed the limit value of 200 Bq/kg [19]. In view of the above, the slag can be used as a building material in buildings intended for the stay of people and livestock. Furthermore, the tested slag can also be used in road, railway and underground construction, because the value of the cosmic radiation dose rate does not exceed the allowed value of 300 nGy/h and it is not necessary to cover the slag in addition with a layer of material with low concentrations of natural radioactive elements [19].

To obtain the desired workability of the HPC mixtures, an effective superplasticizer, CX ISOFLEX 793, based on polycarboxylate ethers with a density of 1.065 g/cm^³^ at 20 °C, was dosed. The water used came from the municipal water supply network.

### 2.2. Mixture Design and Sample Production

The fixed components of the high-performance concrete reference mix (HPC0) were cement, microsilica, gravel, quartz sand, superplasticizer, and water. Gravel coarse aggregate with a 2–16 mm fraction was partially replaced by coal slag with an identical fraction in amounts of 10% wt (HPC1), 20% wt (HPC2), and 30% wt (HPC3) according to the experimental plan [37]. Replacement levels of 10%, 20%, and 30% by weight of natural coarse aggregate with coal slag were selected based on preliminary laboratory trials and previous research. These levels allow for a representative analysis of mechanical and durability behavior while maintaining acceptable workability and cohesion in high-performance concrete mixtures. Dosages greater than 30% were avoided due to a significant decrease in workability, which could affect the quality of the compaction and the structural uniformity. The chosen substitution range offers a practical compromise between performance, sustainability, and ease of implementation in real-world construction scenarios. Silica sand with a maximum grain size of 2 mm was used as the fine aggregate. All aggregates used, sand, gravel, and coal slag, were dried for 24 h in dryers at a constant temperature of 95 °C before each sample series was made. It had an impact on the selection of a higher w/b ratio of 0.48, slightly exceeding the typical values of HPC ratios when using aggregates with natural moisture. The optimal addition of microsilica was determined in previous investigations [38]. To reduce the amount of cement required to produce HPC, condensed microsilica was added in an amount of 5% cement weight. To achieve this, the superplasticizer was dosed based on the weight of cement and microsilica at a rate of 1.5%. The specific formulas for the compounds are given in Table 3.

Four high-performance concrete mixtures were prepared. The first mixture did not contain coal slag. The following three contained coal slag with a percentage ranging from 10% to 30% and with a reduced amount of gravel aggregate by the weight of the added coal slag.

**Figure 3:**
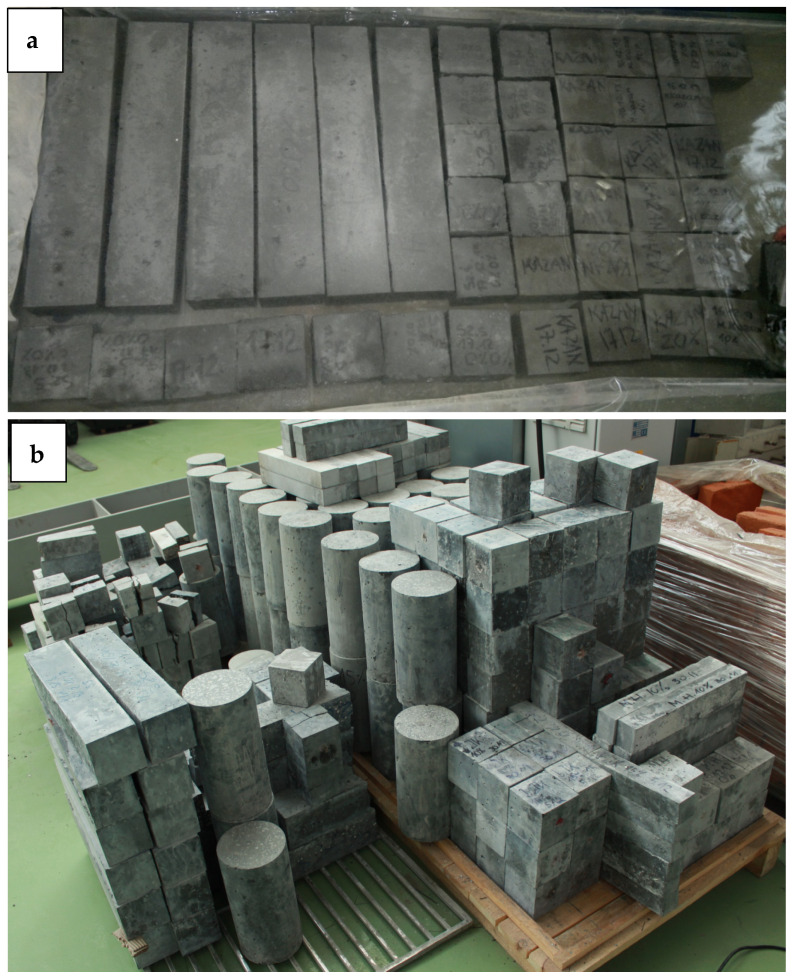
Sample production process: (**a**) curing in water, (**b**) curing at constant room temperature.

High-performance concrete mixtures were produced by mixing dry fine aggregate (quartz sand) and coarse aggregate (gravel and optionally coal slag) for 3 min and then adding half of the tap water. The next stage of mixing the ingredients lasted 2 min. Then, cement, microsilica, the rest of the water, and superplasticizer were added. This mixing phase lasted approximately 3 min. After the mixing process, the molds were filled with the ready-made material, and then the HPC was compacted in layers by means of a vibrator. All samples were protected from the loss of moisture. Until demolition, they were stored for 24 h at a temperature of approximately 23 °C. Then, they were placed for 14 days in a tank of water at 20 °C and 100% relative humidity (Figure 3a). For the remaining time until tests were conducted at 28, 56, and 730 days, the samples were kept at constant room temperature under laboratory air conditions (Figure 3b). At 28 days, the absorbability, density, porosity, compressive strength, flexural strength, tensile splitting strength, and modulus of elasticity were tested. Furthermore, at 56 and 730 days, the strengths, elastic modulus, and ultrasonic pulse velocity (UPV) of HPC were determined. To obtain the various properties of hardened HPC, a test program was carried out using 100 mm cubes, 300 mm high cylinders 150 mm in diameter, and 50 × 50 × 250 mm prisms. For all the characteristics investigated, the measurements were repeated at each point in the experiment plan three times. The average value of the results for HPC evaluation was used. It should be noted that while long-term curing in this study was performed under stable laboratory conditions to ensure the reproducibility of the result, actual construction environments are subject to fluctuating temperature and humidity. Therefore, future investigations will focus on evaluating the durability and performance of high-performance concrete with coal slag under variable field conditions, including drying–wetting cycles, thermal gradients, and seasonal climate exposure.

### 2.3. Research Methodology for Hardened HPC Samples

This study involved a comprehensive testing program to evaluate the physical, mechanical, and microstructural properties of high-performance concrete with varying levels of replacement of coal slag. The tests included determination of density, porosity, water absorption, compressive strength, tensile strength, splitting, flexural strength, static modulus of elasticity, and ultrasonic pulse velocity (UPV). In addition, a FEI Quanta 250 FEG scanning electron microscope (FEI Company, Hillsboro, OR, USA), equipped with an EDAX energy-dispersive X-ray (EDS) analyzer (EDAX Inc., Mahwah, NJ, USA), was used to analyze the microstructure of the hardened concrete.

HPC was subjected to the water absorption test according to the PN-EN 13,755 standard [39] on cuboid samples with a side of 100 mm after meeting the requirement of a curing period of 28 days. The 72-h cube drying was carried out in an electric oven at 105 °C. After being removed, they were cooled in a laboratory for 24 h, then weighed and placed in a tank with water at 20 °C for 30 min. After cube samples were taken from the water, they were dried until the surface was saturated and weighed again. The increase in the mass of the sample after immersion in water was used to determine the water absorption of the sample. At 28 days, the HPC density and porosity were measured in cubes in relation to PN-EN 12390-7 [40].

**Figure 4:**
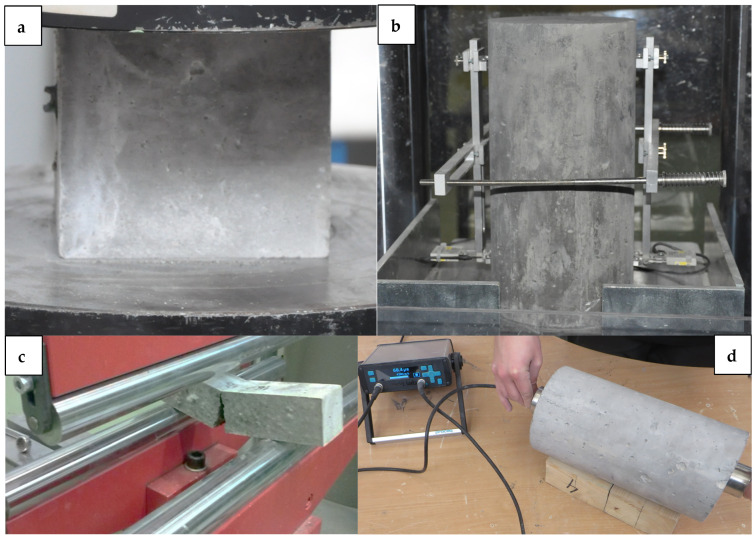
Tests of HPC samples: (**a**) compressive strength, (**b**) modulus of elasticity, (**c**) flexural strength, and (**d**) ultrasonic pulse velocity.

Based on PN-EN 12390-3 [41] and PN-EN 12390-6 [42], compressive strength (Figure 4a) and tensile split tests were performed on cubic samples of dimensions 100 mm × 100 mm × 100 mm. Both strength tests were evaluated at 28, 56, and 730 days.

The modulus of elasticity at compression was determined using three 150 mm in diameter and 300 mm in height cylinders, measuring the displacement within the stress range starting from 0.5 MPa and ending at 30% of the compressive strength for HPC. The examinations were carried out using an extensometer-equipped device to determine the modulus, according to ASTM C469/C469M-14 [43] (Figure 4b).

According to the PN-EN 12390-5 standard [44], the flexural strength of the prismatic specimens 50 mm × 50 mm × 250 mm was used in a three-point bending scheme to determine (Figure 4c). A loading rate of 0.05 MPa/s was applied.

The integrity and homogeneity of HPC were verified using an ultrasonic pulse velocity (UPV) test [45,46]. The UPV test was performed using a UPV tester (Proceq SA, Schwerzenbach, Switzerland) test was carried out on 150 mm diameter and 300 mm height cylinders after 730 days of curing, according to PN-EN 13791 [47] (Figure 4d).

From fragments of undamaged cubes after the tensile splitting strength test, three samples were taken from the exposed surfaces of HPC2 and HPC3 with the highest coal slag content. Analyses of their structure, together with determination of elemental compositions, were performed using an electron microscope with an EDX X-ray dispersion analyzer. The results were developed in the form of microphotographs, spectral diagrams, and tables. On the basis of the analysis of the results, probable causes of improvement in the quality of concrete with the addition of slag were determined.

## 3. Results and Discussion

### 3.1. Absorbability, Density, and Porosity

**Figure 5:**
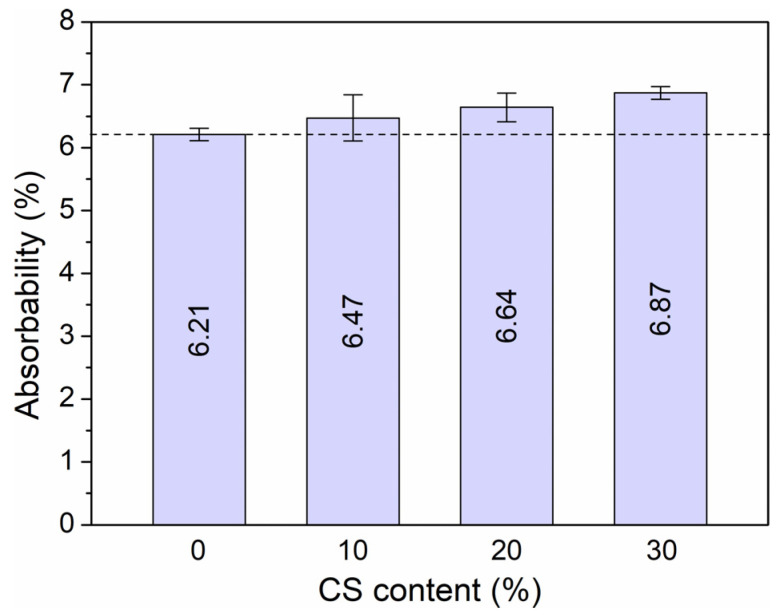
Absorbability of high-performance concrete depending on the addition of coal slag.

**Figure 6:**
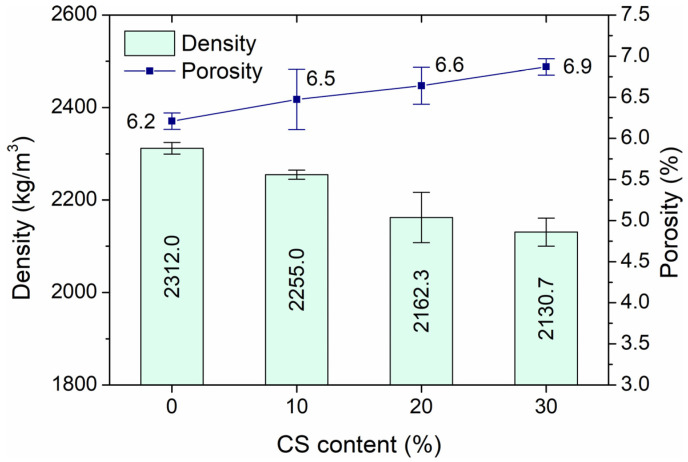
Density and porosity of high-performance concrete depending on the addition of coal slag aggregate.

The results in Figure 5 and Figure 6 show that the amount of coal slag affects a growth in porosity and absorbability and a decrease in HPC density. The density drop is small and amounts to 8% for samples with 30% slag, compared to samples without an additive. Porosity is in the range of 11 to 15% and is slightly higher than typical values for HPC without waste. The water absorption of the samples ranges from 6.21 to 6.87%. The addition of 10% coal slag causes a decrease in water absorption of 4% and a 30% addition of 11% relative to specimens without CS.

The reasons for the increase in the water absorption and porosity of HPC should be the porous structure of the coal slag. Bouzoubaa et al. [48] and Gesoglu et al. [49] found that the high water absorption in slag concrete can be balanced by adding silica fume. Smarzewski and Błaszczyk [7] showed that the addition of cement kiln dust (CKD) to HPC can reduce water absorption, and this effect increases with a higher CKD content. This is due to the filling of the empty spaces by very fine particles of cement dust. On the other hand, Kirgiz et al. [28] reported that the water adsorption of hardened grout composites doped with carbon flakes and colloidal carbon flakes is 10.12%, which is less than 10.3% lower than that of the control grout composite. Lower water adsorption reduces issues such as efflorescence, alkali-silica reaction, and harmful chemical minerals from the Earth. Furthermore, they observed that adding 1 g of carbon flake to the composites reduced the apparent porosity in the range of 8.6 to 22.5%, and adding colloidal carbon flake reduced the apparent porosity by 13.6 to 15.7%, which is caused by the enhanced pozzolanic effect of fuel ash.

Kim and Lee [24] carried out tests on the density of concrete containing bottom ash after 7 and 28 days of curing and found that the density of hardened high-strength concrete decreased linearly with increasing content of coarse bottom ash and did not exceed 2000 kg/m^3^. Syarif et al. [27] noted similar regularities. In the investigation, they noted that the apparent density of fresh concrete with cement made of more than one type of byproduct, such as pulverized fuel ash, bottom ash, calcined clay waste, Mediterranean soil, and household waste ash, was 2081 kg/m^3^, and the dry density 2032 kg/m^3^. These values were significantly lower than in the case of Portland cement concrete density equal to 2525 kg/m^3^. In turn, ref. [7] found that HPC showed a slight increase in density with increasing CKD addition. The highest density of 2244 kg/m^3^ was obtained with a 20% CKD content.

### 3.2. Compressive Strength

**Figure 7:**
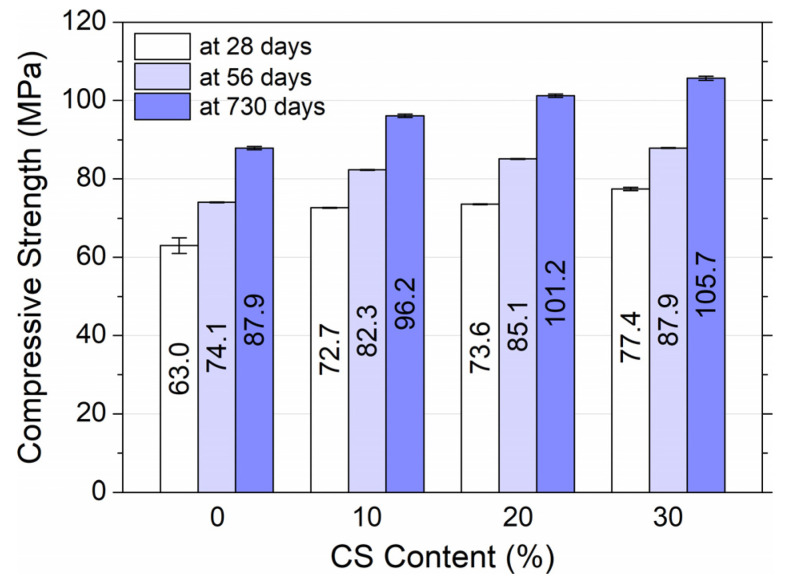
Compressive strength of high-performance concrete depending on the content of coal slag and age of concrete.

Based on the results presented in Figure 7, it was found that the amount of coal slag increased the compressive strength at 28, 56, and 730 days for the HPC samples. For the first series of tests, after 28 days from the molding of the samples, the difference was not significant. For the HPC1 mixture, an average strength of 72.7 MPa was observed, and the strengths of the HPC2 and HPC3 mixtures were higher by 1% and 2.4%, respectively, compared to the concrete with a 10% slag content. After 56 days, an increase in strength of 13.5% was observed for HPC1, 15.8% for HPC2, and 18.1% for HPC3 compared to the strength at 28 days. After 2 years, even higher increases in strength were observed, which were 16.5%, 18.8%, and 19.5%, respectively, compared to strength at 56 days. Based on the above trends, it was found that the addition of coal slag to HPC has a positive effect on the compressive strength of hardened concrete. It should be noted that the highest increase in strength was obtained for HPC with the highest slag content.

The compressive strengths of the normal strength of the alkali-activated coal gangue slag concrete at 28 and 90 days were analyzed by Ma et al. [29], showing that increasing the content of the slag significantly increased the compressive strength. Samples with a higher slag content (10–50%) exhibited increases in compressive strength of 56.1% to 163.8% compared to samples without slag. This study also found that increasing the content of the coarse coal gangue aggregate (CGCA) reduced the compressive strength. However, coal aggregate concrete still outperformed traditional concrete in strength. Calcined CGCA further improved compressive strength compared to raw CGCA, with improvements ranging from 7.6% to 15.5% depending on the slag content. This was attributed to the calcined porous surface of CGCA and reactive SiO_2_ and Al_2_O_3_, which improve bonding with mortar.

The chemical composition of crystallized coal slag is similar to that of the induction furnace slag produced in foundries during the smelting of iron alloys. Based on the results of the research by Bagheri et al. [50] conducted on concrete with the addition of very low reactivity blast furnace slags, it can be concluded that concrete with coal slag will be characterized by a similarly low increase in strength over time. Thomas et al. indicated [22] that the compressive strength of concrete containing 20–25% slag and 3–5% silica fume after 7 days is almost equal to the compressive strength of the control mixture, and after 28 days exceeds it. A similar trend was observed in studies of lightweight concretes with coal slag and the addition of fly ash [23]. The strength of the slag-coated concrete was found to be determined by the quantity and not the quality, of the cement. The reason for this is the low strength of the slag aggregate and the large number of voids that remain to be filled. It was also observed that the strength after 90 days increased by more than 40% compared to the strength at 28 days. Therefore, it was recommended that the strength determined after 90 days be considered as the reliable strength of normal concrete with slag. Syarif et al. [27] performed compressive strength tests for concrete with cement containing pulverized fuel ash, bottom ash, calcined clay waste, Mediterranean soil, household ash, and Portland concrete. Although the strength of the new cement concrete was lower, the rate of increase in its strength was higher than that of the Portland concrete. This was determined by the chemical composition of the new cement, including a higher content of calcium oxides, silicon, iron, aluminum, magnesium, sodium, potassium, and sulfates, and a lower content of gypsum. However, the studies conducted by Kim and Lee [24] suggest that the inclusion of coarse bottom ash had little effect on the compressive strength of the concrete, although this 25% addition slightly increased the strength. In connection with the above results, it can be concluded that HPC with coal slag can be used to place large masses of concrete because of the slower release of heat. Furthermore, because of the relatively low water demand of coal slag, its combination with silica fume can balance the water demand of the HPC mixture that contains only microsilica.

**Figure 8:**
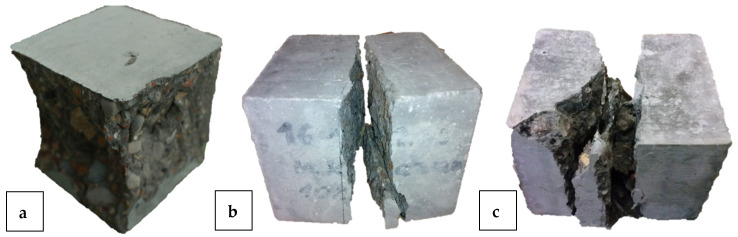
High-performance concrete failure mechanism with 10% coal slag (**a**) after compression at 28 days, (**b**) after splitting tension at 28 days, (**c**) after splitting tension at 56 days.

Coal slag did not change the failure mechanisms of HPC samples under compression stress from standard hourglass-shaped failure modes (Figure 8a). Figure 8b,c shows the failure patterns of high-performance concrete made with 10% coal slag content at 28 and 56 days of curing, respectively. The damage pattern changes slightly with time. After 56 days of curing, the splitting is more irregular, which indicates an increase in the adhesion of the slag aggregate and has an impact on the increase in HPC tensile strength with age.

### 3.3. Flexural Strength

**Figure 9:**
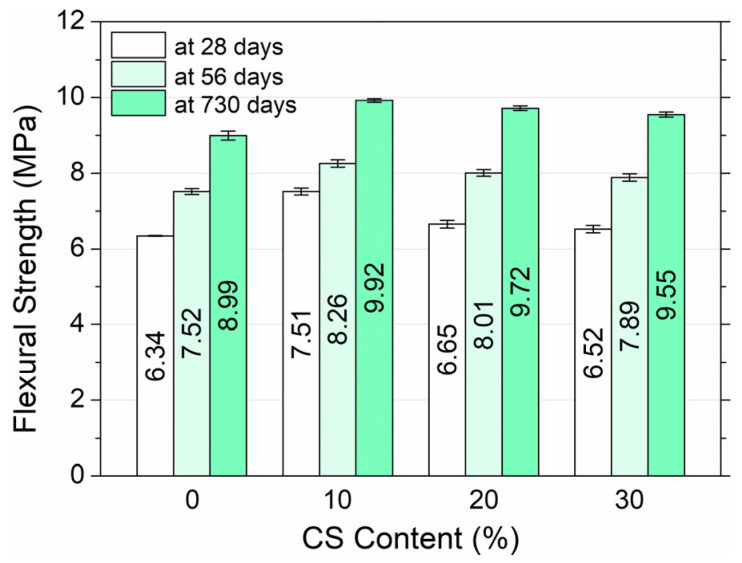
High-performance concrete flexural strength depending on coal slag content and curing age.

Figure 9 shows the effect of coal slag on the flexural strength of HPC after 28, 56, and 730 days of sample maturation.

Similarly to the compressive strength of HPC, the flexural strength increased with the addition of CS. At 28 days, the flexural strength of the control mix HPC0 (0% CS) was 6.34 MPa, while for HPC1, HPC2, and HPC3 it reached 7.51, 6.65, and 6.52 MPa, respectively. Increases of 18.5%, 4.9%, and 2.8% were recorded compared to the strength of HPC0 concrete. Flexural tensile strength also increased with age. Between 28 and 730 days, the control concrete HPC0 showed an increase of 41.8%, while for HPC1 it increased 32.1%, for HPC2 it increased 46.2%, and for HPC3 it increased 46.5%. The greatest increase in flexural strength was observed over time for HPC3 with 30% added coal slag. The main reason is probably that the craters and the large pores on the surface of the coal slag are filled with cement paste. Cracks were found to spread through rough coal slag and smooth gravel aggregate grains due to the low strength of the coarse natural and waste aggregate. Compared to other studies, Park et al. [51] found that the addition of slag had little effect on the flexural strength of plain concrete.

### 3.4. Splitting Tensile Strength

**Figure 10:**
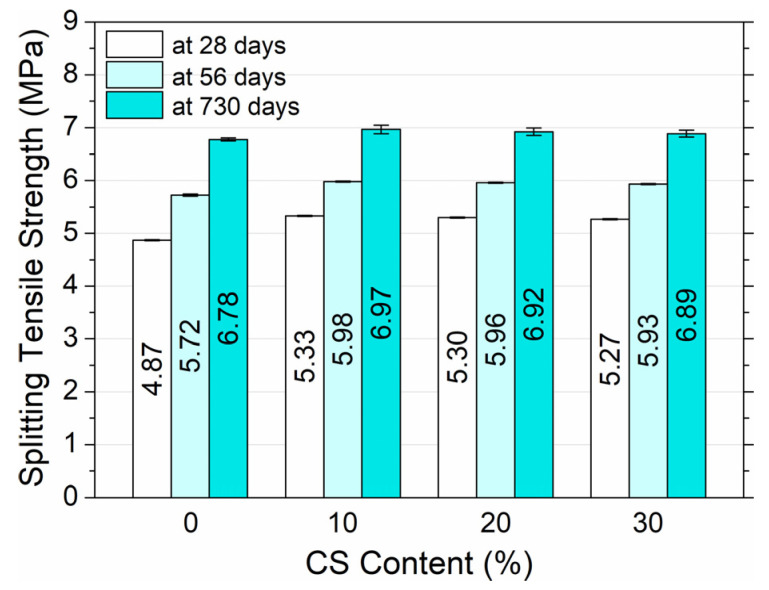
Splitting tensile strength of high-performance concrete depending on CS content and curing age.

The summary of the HPC tensile strength splitting results according to the maturation time is presented in Figure 10.

The variation in the splitting tensile strength with the addition of CS was similar to that observed for the flexural strength. The splitting tensile strength of HPC increased with the addition of CS. After 28 days, the tensile strength of the concrete HPC0 (0% CS) was 4.87 MPa and HPC1 (10% CS), HPC2 (20% CS) and HPC3 (30% CS) reached 5.33, 5.3 and 5.27 MPa, respectively, which meant that the strength increased by 9.4%, 8.8% and 8.2% compared to the strength of the reference concrete HPC0 (0% CS). The tensile strength of the concrete split was found to increase with age, regardless of the slag content. At 56 and 730 days, HPC0 (0% CS), HPC1 (10% CS), HPC2 (20% CS) and HPC3 (30% CS) reached strengths of 5.72 and 6.78, 5.98 and 6.97, 5.96 and 6.92, 5.93 and 6.89 MPa, respectively, which meant strength increases in the range of 17.5–39.2%, 12.2–30.8%, 12.5–30.2% and 12.5–30.7% compared to the 28-day strength. With a maturation time of 28 to 730 days, the percentage increase in HPC tensile splitting strength for the reference mixture (without CS) was 39.2%, for HPC1—30.8%, for HPC2—30.2%, and for HPC3—30.7%. The addition of 10–30% slag increased the HPC tensile splitting strength by 8–9% after 28 days and by 2–3% after 730 days, probably due to filling the craters and pores with cement paste. The rough surface of the slag also promoted the formation of a stronger bond at the interface between the aggregate and the cement mortar, which consequently affected the inhibition of crack propagation to some extent. As a result of the mechanisms involved, HPC with the addition of coal slag is more resistant to splitting and bending.

Syarif et al. [27] observed that ordinary concrete made with new cement based on the burning of the quinary byproduct after drying curing shows a higher tensile splitting strength than that treated with water curing. Furthermore, the results of the tensile splitting strength of the new cement concrete and the Portland cement concrete were very close to each other. Although the new cement was finer than Portland cement, this was not a factor that positively affected the splitting tensile strength of the concrete.

**Figure 11:**
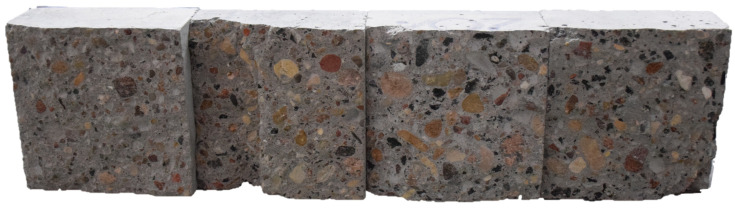
High-performance concrete samples with different coal slag content, from left to right: 0%, 10%, 20%, 30%.

The cross sections of the samples after splitting were subjected to macroscopic evaluation. In the attached photograph (Figure 11), no agglomeration was observed in the macrostructure of the material, indicating the precise selection of the mixing time, which ensured the homogeneous consistency of the mixture, influencing the relatively uniform morphology of the samples. It can also be concluded that the vibration and laying process of the high-performance concrete mixture was properly coordinated. No visible delamination or segregation of the aggregate was observed in the photos. This indicates the proper and uniform distribution of coal slag in the high-performance concrete mixture, which was not associated with a change in HPC color. Therefore, it was confirmed that the HPC production process was carried out in accordance with the assumed procedures, which contributed to obtaining homogeneous material without delamination, agglomeration, or sedimentation of the components.

**Figure 12:**
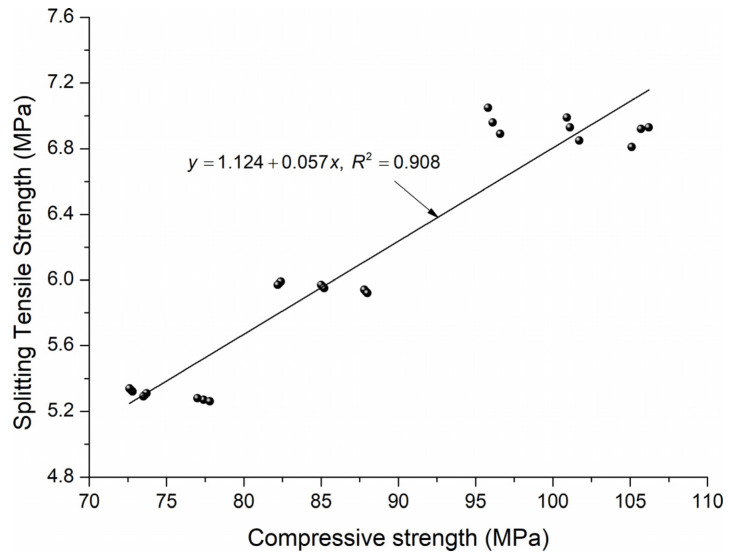
Correlation between compressive strength and splitting tensile strength of HPC with 10–30% coal slag.

The relation between compressive strength after the results of the tensile splitting strength test and the volume content of the coarse coal slag aggregate was determined in Figure 12. This correlation can be described by the following equation: $f_{ct,spl}=1.124+0.057f_{c}$. The linear trend was characterized by a strong correlation coefficient $R^{2}=0.908$ and very low intercept errors. The proposed equation can be used successfully to predict the relationship between the compressive strength of high-performance concrete and the tensile splitting strength for the content of coal slag between 10 and 30%.

**Figure 13:**
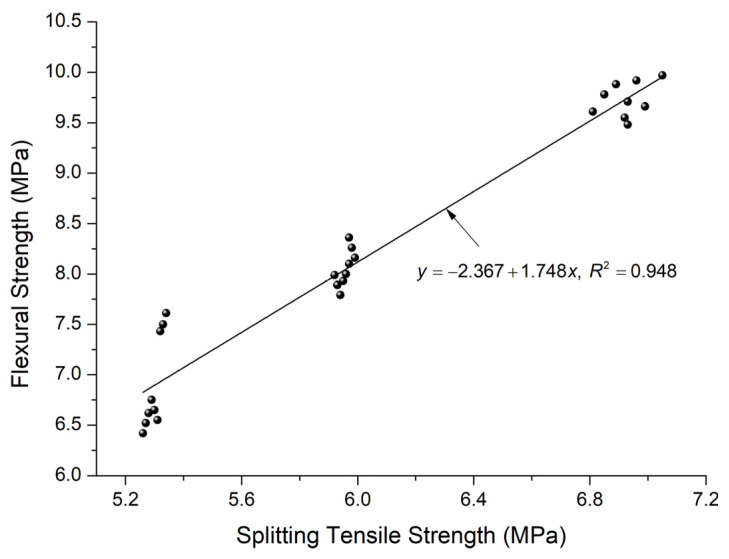
Relationship between splitting tensile strength and flexural strength of HPC with 10–30% coal slag.

Figure 13 presents the relationship between the tensile splitting strength and the flexural strength of three types of high-performance concrete with 10–30% additions of coal slag aggregate.

A linear $f_{ct,fl}=-2.367+1.748f_{ct,spl}$ relationship of the form *y* = *b* + *ax* appears to best fit the data, *R*^2^ = 0.948. The literature states that the model can be considered correct when the *R*^2^ coefficient is greater than or equal to 0.7 [52]. Consequently, the proposed equation can be used successfully to project the relationship between the split tensile strength and the flexural strength of high-performance concrete containing coal slag.

### 3.5. Modulus of Elasticity

**Figure 14:**
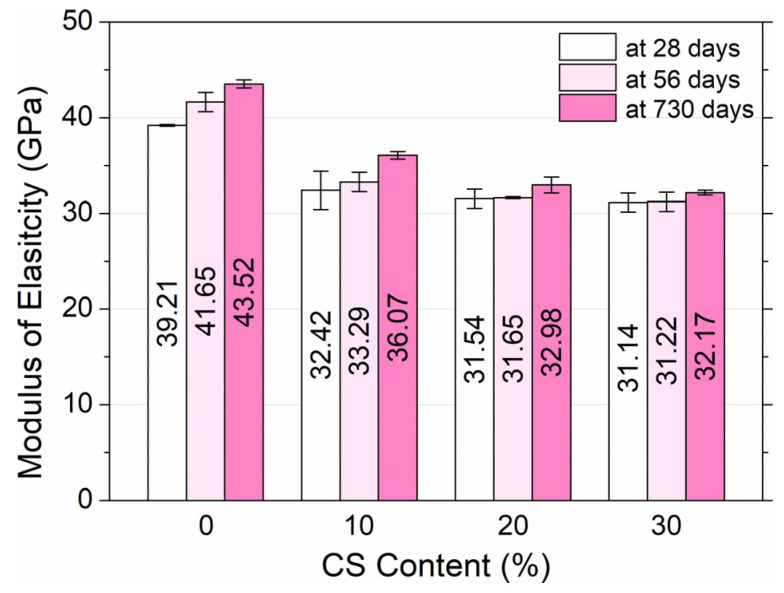
Modulus of elasticity of high-performance concrete in dependence on the content of coal slag and age of concrete.

The static modulus of HPC elasticity was calculated for 0.33 of the maximum loads during compression of cylindrical samples. The elastic modulus of high-performance concrete was determined at 28, 56, and 730 days, and the results are revealed in Figure 14.

The HPC elastic modulus with 10% coal slag content and above was significantly lower than the elastic modulus of the reference concrete. Measurements demonstrate that the higher the content of CS, the lower the modulus of elasticity. HPC0 exhibited an elasticity modulus at 28 days of 39.21 GPa. HPC1, HPC2, and HPC3 obtained moduli of 32.42, 31.54, and 31.14 GPa, respectively. Modulus drops were 20.9% (HPC1), 24.3% (HPC2), and 25.9% (HPC3) compared to HPC0. In turn, the 56 and 730 day moduli of the control HPC were 41.65 and 43.52 GPa, and high-performance concrete without CS achieved modulus increases of 6.2–11.0% during this period of time. Increases in moduli were also observed in 10% CS—2.7–11.3%, 20% CS—0.3–4.6%, and 30% CS—0.3–3.3%. We should note that the greatest impact on this parameter, an increase of 11.3%, was achieved during the long maturation time, at 730 days, for HPC1. This increase decreased with a larger addition of slag. The internal porosity of the slag was found to be the main factor that caused the elastic modulus to decrease compared to the reference HPC. The conclusions are consistent with the results obtained by other researchers. Kim and Lee [24] observed that replacing the aggregate with the bottom ash leads to a significant decrease in the elastic modulus of the concrete.

Although the SEM analysis in Section 3.7 shows a partial filling of the coal slag pores with hydration products such as CSH and CH, this refinement is localized and does not fully compensate for the inherent low stiffness and high porosity of the coal slag particles. As a result, the overall elastic modulus of the composite remains lower than that of the reference concrete with dense gravel aggregate. This distinction between local densification and global stiffness explains the observed reduction in modulus despite microstructural improvements.

### 3.6. Ultrasonic Pulse Velocity

**Figure 15:**
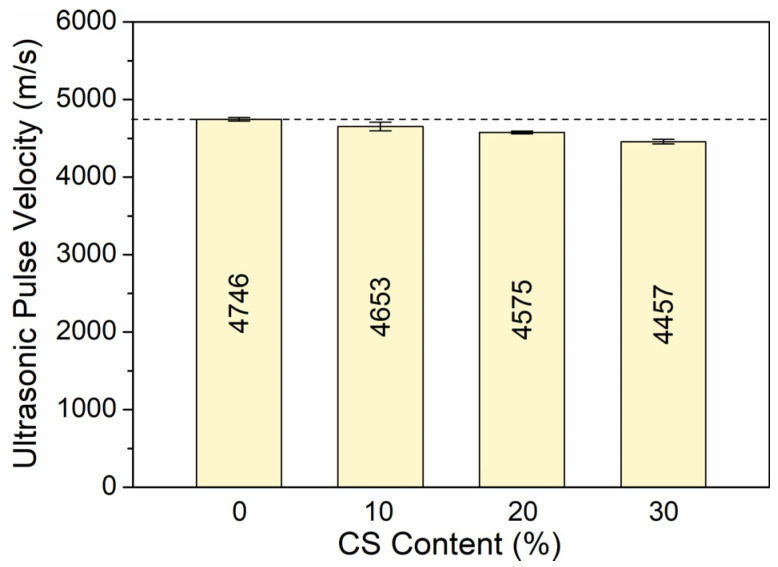
UPV of high-performance concrete depending on coal slag content.

The quality of HPC was determined by a non-destructive ultrasonic pulse velocity (UPV) test using a direct method. A direct pulse transit time was calculated between the two opposite transducers, emitter and receiver, by passing the ultrasonic signal from one transducer to the other. The propagation of the signal was observed directly, and the velocity was determined by measuring the distance between the transducers. The pulse time taken depends on the uniformity and porosity of the HPC, as well as the presence of microcracks [7,45,46]. From the pulse transit time, the velocities were calculated by dividing the pulse distance by the transit time. The test was carried out in all three cube sample planes for each HPC mixture to obtain the mean value of the stable pulse time. The test is used to determine the quality, uniformity, and compressive strength of concrete. The higher the strength and density of concrete, the shorter the time it should take to pass through the sample, and, ultimately, it should increase the wave velocity. Therefore, the compressive strength is directly related to the wave velocity in the concrete specimen. Figure 15 shows the UPV of high-performance concrete containing coal slag after two years of maturation.

From the results, it was observed that the addition of coal slag decreases the velocity of the signals and that CS reduces its value due to the porous nature. Despite this, all mixtures up to 30% CS showed good quality HPC (velocities are greater than 4000 m/s) [53]. The greater the amount of coarse CS aggregate added, the longer it took the waves to pass through the HPC body. After 730 days of maturation, HPC0 without CS reached the highest UPV of 4746 m/s, while the HPC1, HPC2, and HPC3 concrete achieved UPV of 4653, 4575, and 4457 m/s, respectively. The decreases in UPV were 2.0%, 3.7%, and 6.5%, respectively, compared to the UPV of HPC0. This was due to the increase in porous nature and the voids developed as a result of the coal slag. Despite the higher porosity and lower density than control concrete, HPC with the addition of coal slag showed higher compressive strength, tensile splitting strength, and flexural strength. Ultimately, CS did not reduce HPC quality compared to the reference mixture, because the high roughness of the aggregate favored the formation of a strong bond between the slag and the cement paste, which led to the inhibition of microcrack development.

### 3.7. Microstructure

**Figure 16:**
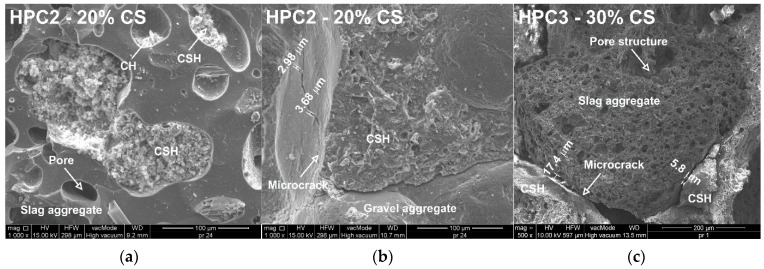
SEM micrographs showing the interfacial transition zone (ITZ) and bond characteristics in high-performance concrete (HPC) with coal slag (CS) aggregate: (**a**) HPC2 with 20% CS—good bond between cement paste and slag ag-gregate, rough slag surface enhancing adhesion; (**b**) HPC2 with 20% CS—weaker bond between gravel aggregate and cement paste, presence of microcracks (2–4 µm) along the interface; (**c**) HPC3 with 30% CS—well-bonded slag aggregate with dense cement paste and rough surface, contributing to higher mechanical strength.

In HPC microstructure analysis, samples taken from undamaged cube parts from the tensile strength test were used. Scanning images and energy-dispersive X-ray spectroscopy (EDX) analyses were performed. Figure 16a shows a microphotograph of the slag aggregate with partially filled voids by large particles of crystallized calcium silicate hydrate (CSH) and occasionally portlandite (CH) in HPC2 with 20% slag. Subsequent microphotographs (Figs. 16b, 16c) show the contact zones of gravel and slag with cement paste for hardened HPC2 with 20% and HPC3 with 30% coal slag, respectively.

**Figure 17:**
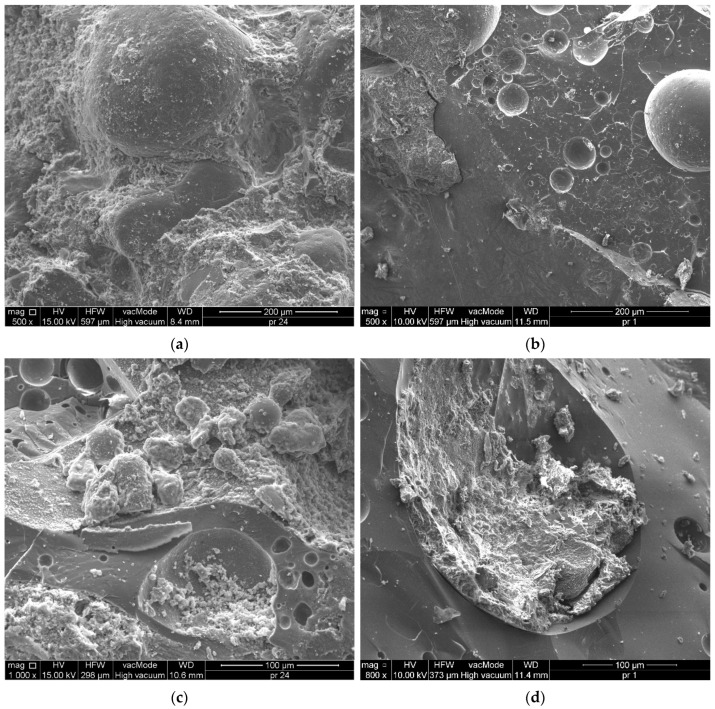

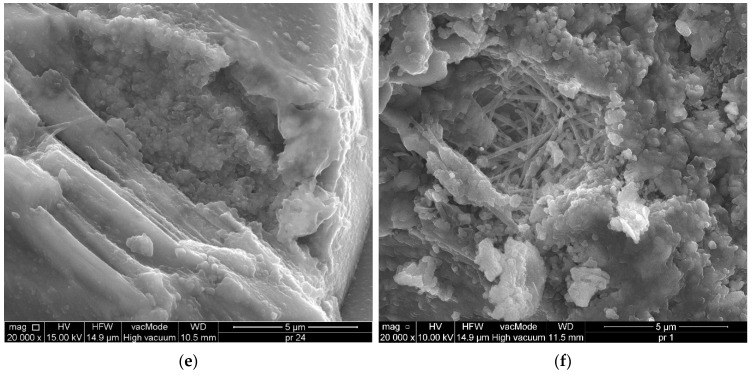
SEM microphotographs of high-performance concrete (HPC) with coal slag. (**a**) HPC2 with 20% CS, magnification 500×. (**b**) HPC3 with 30% CS, magnification 500×. (**c**) HPC2 with 20% CS, magnification 1000×. (**d**) HPC3 with 30% CS, magnification 800×. (**e**) HPC2 with 20% CS, magnification 20,000×. (**f**) HPC3 with 30% CS, magnification 20,000×.

Coal slag is a porous and dry aggregate that intensively draws water from the cement paste, resulting in cracking of the bond between the paste and the aggregate. The structure of the transition zone in high-performance concretes with coal slag was found to be different from that of traditional concrete. Based on SEM microphotographs (Figure 16a–c), a good bond between cement paste and slag aggregate was found to determine the higher strength of HPC compared to reference high-performance concrete with only gravel aggregate. The surface of the slag is rough and irregular, resulting in greater adhesion to the cement paste compared to the gravel aggregate. Furthermore, the bond between the gravel and the paste was weaker, which was confirmed by microcracks with widths of 2–4 µm, which passed through the gravel aggregate and also developed between the paste and the gravel grain. In Figure 17a,b, it can be seen that the samples tested with 20% and 30% slag addition had a uniform structure, and the matrix density increased with increasing slag addition. Furthermore, as a result of the large amount of cement and the increased pozzolanic reaction, refinement processes of the pore microstructure occurred, consisting of partial sealing of the porous structure of coal slags (see Figure 17c,d). With an increasing addition of slag, large particles of a well-crystallized CSH phase could be observed, which contributed to the increase in HPC strength (Figure 17e,f).

Adegoke and Ikumapayi [54] evaluated the microstructure and morphology of the hydration products at 28, 56, and 90 days for concrete with an addition of 0%, 25%, and 45% of slag in the induction furnace using scanning electron microscopy with energy dispersive spectroscopy (EDS). Researchers identified large particles of crystallized CSH. They found that the tested samples had a more uniform matrix with less slag addition. However, at 45% addition, it was observed that the microstructure was loose and suspension components formed due to reduced cement content and consequently a less pozzolanic reaction. With increasing concrete age, after 90 days of curing, a greater densification of the microstructure was observed, which was justified by the refinement of the pore microstructure over time due to delayed pozzolanic reactions indicated by the decrease in the amount of calcium in the concrete with increasing slag content. XRD patterns showed that changes in the compressive strength of the concrete were initiated with the addition of variable slag in the amorphous part of the structure.

**Figure 18:**
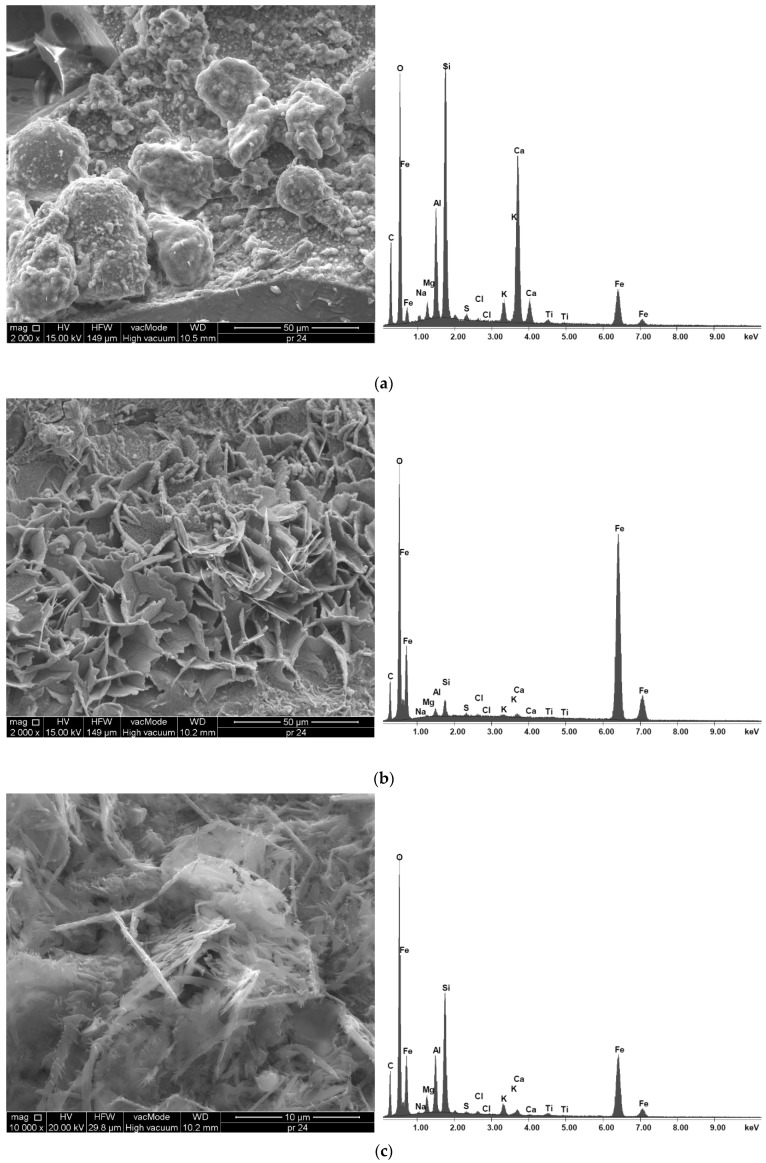

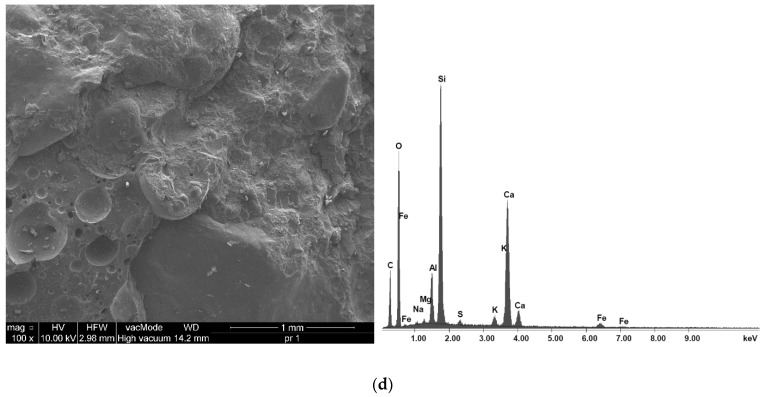
EDX analysis of HPC mixtures using 20% and 30% CS. (**a**) HPC2_1 using 20% coal slag. (**b**) HPC2_2 using 20% coal slag. (**c**) HPC2_3 using 20% coal slag. (**d**) HPC3 using 30% coal slag.

The energy dispersive X-ray spectroscopy (EDX) data shown in Figure 18 and Table 4 provide information on the elements present in a particular mixture, as indicated by the peaks of these elements. The representation of the number of counts is shown along the *y*-axis of the graphs, whereas the energy of the X-rays is shown along the *x*-axis.

Figure 18a shows the composition of the sample, which includes the elements calcium (Ca), carbon (C), silicon (Si), iron (Fe), aluminum (Al), oxygen (O), sodium (Na), magnesium (Mg), sulfur (S), chlorine (Cl), potassium (K) and titanium (Ti), indicating their presence in the material. It should be noted that the high carbon content recorded in all samples is due not only to its presence in the slag aggregate used but also to the pre-spraying process of all samples with carbon during their preparation for SEM analysis. The HPC2_1 sample has a high concentration of Si, which is confirmed by the dominant peak in the EDX analysis. The composition of the HPC2_1 sample with 20% coal slag follows the order of carbon > silicon > calcium > aluminum > iron > potassium > magnesium > sodium > sulfur > titanium > chlorine, as shown in Figure 18a and Table 4, indicating the highest intensity for elements C, Si, Ca, Al and Fe. The SEM images in Figure 18b,c reveal a very distinct development of iron-based mineral bonds in the HPC2 mixture. These enhanced bonds are the key factor contributing to the exceptional compressive strength exhibited by the HPC2 mixture with 20% bottom slag. The composition of the HPC3 mixture with 30% bottom slag follows the order of carbon > silicon > calcium > aluminum > iron > potassium > magnesium > sulfur > sodium. It can be seen that the order of the dominant elements is preserved, but trace amounts of titanium or chlorine are not observed. Figure 18d shows the highest peak intensity of silicon, calcium, and aluminum. The SEM image indicates a higher degree of bond formation in the 20% coal slag scenario compared to the HPC2 mixture. Furthermore, the microphotograph shows that a more densely packed microstructure is obtained in the HPC3 scenario.

The EDX results for HPC shown in Table 4 illustrate the effect of the addition of 20% CS on microstructure compared to the addition of 30% CS. The value of the Ca/Si ratio reflects the more favorable rate of the pozzolanic reaction in HPC3. The results confirm the more active role of silica in strengthening the microstructure in concrete with 30% CS. It can be seen that the calcium content in the HPC3 mixture is higher than in HPC2, indicating its faster set time. In general, these results show that the interaction of the clinker phases with 20% coal slag was less intense compared to the mixture with 30% CS. It was also found that the slightly higher alkali content in HPC2 could have resulted in an increase in the solubility of the clinker phases compared to HPC with 30% CS.

The observed improvements in compressive, tensile, and flexural strength with increasing coal slag content can be attributed to several interrelated microstructural mechanisms. First, the rough and porous surface of the slag particles enhances mechanical interlocking and chemical bonding at the interfacial transition zone (ITZ), as evidenced by the SEM images. Second, the pozzolanic reaction, indicated by the decreasing Ca/Si ratio in EDX results, leads to secondary formation of CSH, which densifies the matrix and fills the voids, reducing the propagation pathways of microcracks. Third, gradual sealing of internal slag porosity contributes to a more compact structure, partially compensating for the initial higher overall porosity. These mechanisms collectively explain the observed long-term strength gain and the slight reduction in the elastic modulus and UPV due to the residual porous nature of the aggregate. Therefore, the microstructural analysis supports a mechanistic understanding of the material behavior and underscores the practical feasibility of using coal slag in high-performance concrete.

## 4. Conclusions

This study demonstrates that replacing up to 30% of coarse natural aggregate with coal slag in high-performance concrete provides clear environmental and engineering benefits, maintaining excellent mechanical and microstructural performance after 730 days of curing. Increasing the content of coal slag resulted in significant long-term improvements in compressive, flexural, and splitting tensile strength compared to the reference mixture. SEM/EDX analyses confirmed excellent interfacial bonding between the slag particles and the cement matrix, despite a moderate increase in porosity and water absorption. Beyond reporting standard performance metrics, this research establishes practical strength correlations (for example, between compressive and tensile strength) that can inform structural design using porous alternative aggregates. Although some findings are consistent with earlier studies, the results offer new insights specific to HPC with coal slag, particularly under curing conditions that simulate real-life structural aging.

On the basis of the analysis, the following conclusions are drawn:

- Porosity and water absorption increase with higher coal slag content. For HPC with 30% CS, porosity and absorption increase by 10.6% and 11.3%, respectively, compared to the reference mix, while the density decreases by up to 7.8%.
- Compressive strength improves significantly with the addition of CS. After 28 days, strength increases range from 15.4% (10% CS) to 22.9% (30% CS), and after 730 days, from 9.4% to 20.2%.
- The flexural and splitting tensile strengths also benefit from CS incorporation, showing consistent growth over time. The splitting tensile strength increases by 8–9% at 28 days and increases by 2–3% at 730 days. Flexural strength improves, especially in the early curing stage, most notably at 10–20% CS.
- Statistical relationships are established between compressive, splitting tensile, and flexural strength, allowing performance prediction for mixes with coal slag aggregates.
- The modulus of elasticity decreases both with time and with increasing slag content, which is attributed to the lower stiffness and higher porosity of coal slag compared to natural aggregates.
- Ultrasonic pulse velocity (UPV) values slightly decline with increased CS content but remain within acceptable ranges for good-quality concrete.
- SEM/EDX microstructural analysis confirms that hydration products (CSH, CH) partially fill the pores of slag grains and contribute to matrix densification, improving overall cohesion and strength development.

Future research should include the assessment of CS-HPC under environmental exposure conditions (e.g., freeze–thaw, chloride ingress), long-term durability modeling, and life-cycle analysis. It is also worth exploring synergies between coal slag and other supplementary cementitious materials for an optimized mix design.

## Figures and Tables

**Table 1:** Technical parameters and chemical composition of binding components.

Material Characteristics	Cement	Microsilica
Specific surface area (m^2^/kg)	398.5	17,000
Water demand (%)	28	—
Start of setting (min)	190	—
End of setting (min)	250	—
Volume stability acc. to Le Chateliere (mm)	0	—
Compressive strength at 2 days (MPa)	30.4	—
Compressive strength at 28 days (MPa)	59.2	—
Flexural strength at 2 days (MPa)	5.4	—
**Composition (%)**		
SiO_2_	20.46	94.80
Al_2_O_3_	4.15	1.30
Fe_2_O_3_	3.37	0.83
CaO	65.10	0.56
MgO	1.21	0.71
SO_3_	2.58	—
K_2_O	0.46	1.26
Na_2_O	0.24	0.41
Cl	0.09	—
Loss in ignition	3.44	0.12
Insoluble matter	0.29	—

**Table 2:** Natural radioactive isotope concentrations for the slag sample.

Sample Description	Concentrations of Natural Radioactive Isotopes (Bq/kg)			Activity Indicators		Gamma Radiation Dose Rate Value
	Potassium K-40	Rad Ra-226	Thorium Th-228	f_1_	f_2_ (Bq/kg)	nGy/h
Coal slag	565 ± 60	122.1 ± 15.1	99.4 ± 8.9	1.09 ± 0.05	122.1 ± 15.1	142.4

**Table 3:** High-performance concrete mix proportion per 1 m^3^.

Mix ID	Coal Slag		Aggregate		Cement (kg)	Water (L)	Microsilica (kg)	SP (kg)
	Portion (%)	Weight (kg)	Sand (kg)	Gravel (kg)				
HPC0	0	–	539	1229	372	186	18.6	5.9
HPC1	10	123		1106				
HPC2	20	246		983				
HPC3	30	369		860				

**Table 4:** EDX analysis of HPC.

Formula	Atom [%]			
	HPC2_1 20% CS	HPC2_2 20% CS	HPC2_3 20% CS	HPC3 30% CS
Calcium	6.43	0.23	0.32	7.04
Carbon	32.35	26.04	29.64	31.81
Silicon	9.15	1.37	7.60	11.95
Iron	2.93	27.12	8.02	0.49
Aluminum	3.87	0.76	3.78	2.47
Oxygen	42.98	43.72	48.03	45.11
Sodium	0.40	0.00	0.17	0.20
Magnesium	0.72	0.21	1.41	0.23
Sulfur	0.18	0.18	0.09	0.23
Chlorine	0.07	0.14	0.16	—
Potassium	0.73	0.14	0.60	0.48
Titanium	0.18	0.07	0.18	—
Calcium/Silicon ratio	0.70	0.17	0.04	0.59

## Data Availability

The raw data supporting the conclusions of this article will be made available by the authors on request.
